# Immunophenotypic challenges in diagnosis of CD79a negativity in a patient with B acute lymphoblastic leukemia harboring intrachromosomal amplification of chromosome 21: a case report

**DOI:** 10.1186/s13256-021-03128-2

**Published:** 2021-10-28

**Authors:** A. Berhili, M. Bensalah, J. ElMalki, A. Elyagoubi, R. Seddik

**Affiliations:** 1 Hematology Laboratory, Mohammed VI University Hospital, Oujda Universite, BP 4806, 60049 Oujda, Morocco; 2 Genetics Unit, Faculty of Medicine and Pharmacy, Mohammed Premier University, Oujda, Morocco

**Keywords:** Flow cytometric immunophenotyping (FCI), B-cell acute lymphoblastic leukemia, iAMP21, CD79a

## Abstract

**Background:**

Being expressed in all stages of B-cell development and having a significant value on the European Group for the Immunological Characterization of Acute Leukemias scoring system, CD79a is considered as an excellent pan-marker for lineage assignment of B cells by flow cytometry. Therefore, any lack or decrease in CD79a expression makes the diagnosis of B acute lymphoblastic leukemia cases very challenging, especially in developing country laboratories where flow cytometry analyses are not always available and, when they are, they are limited in the number of markers used for lineage assignment. Since this case is potentially interesting, we report a B acute lymphoblastic leukemia case with a lack of expression CD79a associated with intrachromosomal amplification of chromosome 21 genetic abnormality. We further discuss the practical challenges in the diagnosis of this case.

**Case presentation:**

We present the case of an 8-year-old Caucasian boy from eastern Morocco who was initially hospitalized for a hemorrhagic syndrome. Peripheral blood smear examination showed a significant number of blasts suggesting acute leukemia. Bone marrow was studied for morphology, cytochemistry, immunophenotyping, and cytogenetics. Flow cytometry analyses showed expression of CD19, CD22, CD10, CD34, and HLA-DR markers by leukemic blasts. The expression of CD79a, which was checked with two different monoclonal antibodies, confirms that this marker was severely decreased in this case. Cytogenetic study performed by fluorescence *in situ* hybridization revealed the presence of intrachromosomal amplification of chromosome 21, a cytogenetic abnormality that is specific for B acute lymphoblastic leukemia.

**Conclusion:**

CD79a is one of the critical markers in the assignment of B acute lymphoblastic leukemia. In our case, we were lucky enough to be assisted by a few other markers of the B lineage that were positive in this case. Also, we mention the importance of proceeding to cytogenetic study, which in our case helped us to confirm the diagnosis made by flow cytometry by highlighting a cytogenetic abnormality that is specific to B acute lymphoblastic leukemia.

## Introduction

The study of expression of the mb-1 (CD79a) chain is of significant interest in B acute lymphoblastic leukemia (B-ALL) lineage assessment. Alongside B29 (CD79b), it constitutes the CD79 molecule. In association with surface immunoglobulin (sIg), CD79 constitutes the B-cell antigen receptor complex and plays a critical role in B-cell maturation and activation [1]. The CD79a (mb-1) gene products are B-cell-specific essential components of the B-cell receptor (BCR) and are expressed at practically all stages of B-cell differentiation [2, 3]. This antigenic marker constitutes a very valuable pan marker in the diagnosis of B acute lymphoblastic leukemia.

Because of its high degree of specificity for B-cell lineage differentiation, CD79a was considered as the B marker cell equivalent of CD3 by many authors [4, 5]. The European Group for the Immunological Characterization of Acute Leukemias (EGIL) selected CD79a as one of the most potent markers for B-lineage assessment in its proposed immunological classification table of acute leukemias. This marker was given the highest score (2 points) compared with the CD19 marker considered as the key marker for the B-lineage assessment (1 point) [6]. Awareness of the importance of the CD79a marker for the development of B lymphocytes and its significance in EGIL classification makes assessment of B-ALL very challenging in cases of complete or partial lack of expression of this marker. In this report, we described a case of B-ALL showing complete loss of expression of CD79a associated with intrachromosomal amplification of chromosome 21 (iAMP21). To the best of our knowledge, this is a rare report of a B-ALL case with a genetic abnormality in which there is a complete loss of CD79a expression. We further discussed the practical challenges in the diagnosis and monitoring of this case.

## Case presentation

An 8-year-old Caucasian boy from eastern Morocco was hospitalized at the Mohammed VI University Hospital. He was initially admitted for a hemorrhagic syndrome with multiple ecchymoses stretching on all four limbs and face, with petechiae in his ear without infection or anemic syndromes or notion of motivating trauma. Physical examination was otherwise unremarkable. Complete blood cell count was done using automated hematologic analyzer (Sysmex, XN1000, Kobe, Japan). Smear examination of peripheral blood (PB) showed significant number of blasts suggestive of acute leukemia. Bone marrow smears were stained with May–Grünwald Giemsa and analyzed according to routine clinical laboratory procedures. The cytochemical staining was performed for myeloperoxidase (MPO) activity of blasts using Myeloperoxydase Kit M (RAL Diagnostics, France).

**Fig. 1:**
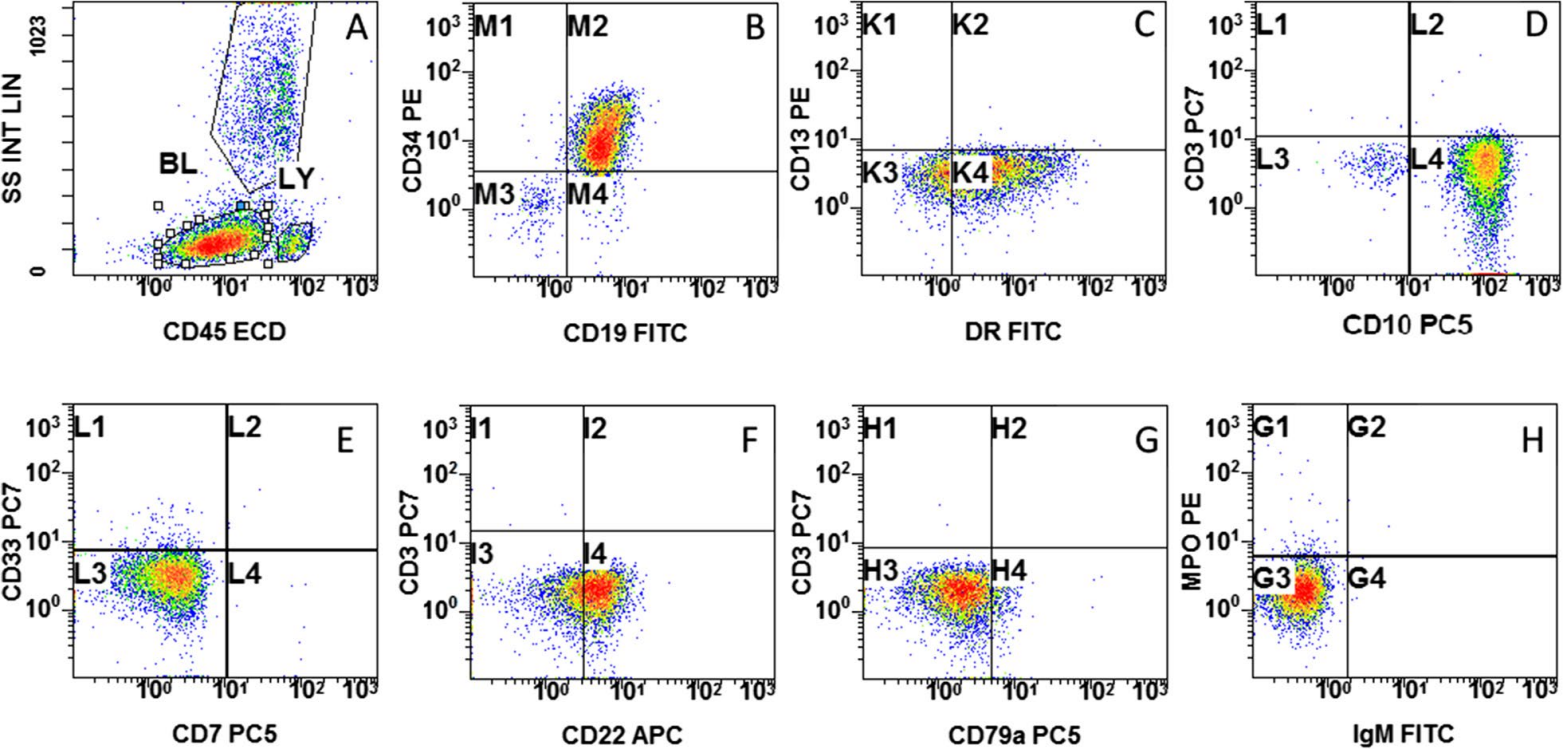
Flow cytometric immunophenotyping graphs. BL, blasts; LY, lymphocytes. **A** SSC and CD45 characteristics of leukemic cells analyzed; **B** expression of CD19 and CD34; **C** expression of HLA-DR and lack of expression of CD13; **D** strong expression of CD10 and lack of expression of sCD3; **E** lack of expression of CD13 and CD7; **F** expression of CD22 and lack of expression of cCD3; **G** lack of expression of CD3 and CD79a; **H** lack of expression of MPO and IgMc

Flow cytometric immunophenotyping of bone marrow aspirate (BMA) was processed using no-wash procedures and via the use of a comprehensive six-color antibody panel for acute leukemia on a Navios flow cytometer (Beckman Coulter, FL, USA). This panel included monoclonal antibodies against CD3, CD10, CD13, CD7, CD19, CD22, CD33, CD34, CD45, CD117, HLA-DR, cytoplasmic CD79a (monoclonal CD79a; pc5, clone HM47), cytoplasmic CD3, cytoplasmic immunoglobulin (Ig)M, and cytoplasmic MPO. For intracellular antigen evaluation, we used PerFix-nc, no centrifuge assay kit (Beckman Coulter, FL, USA). For surface antigen evaluation, we used VersaLyse lysing solution (Beckman Coulter, FL, USA), as per manufacturer’s guidelines. Data collection and analysis was done on the software that manages the Navios flow cytometer version 1.3 using a CD45–side scatter-based gating strategy (Fig. 1). Erythrocytes, platelets, dead cells, and debris were excluded on the basis of CD45 staining and side scatter characteristics. The discriminating boundaries between the positive and negative fluorescent regions was established using autofluorescence and isotypic controls. The results were expressed as percentage of cells in the positive region.

To complete this study with cytogenetic analysis and aiming to review this case with a larger B-ALL panel with another CD79a clone, this BMA was sent to an independent specialized laboratory. The immunophenotyping of BMA in this laboratory was performed using a panel of acute leukemia-containing monoclonal antibodies against CD1a, CD3, CD7, CD5, CD10, CD13, CD38, CD11c, CD19, CD20, CD22, kappa and lambda light chains, CD33, CD34, CD14, CD45, CD36, CD117, HLA-DR, cytoplasmic CD79a, cytoplasmic CD3, and cytoplasmic MPO. Analyses was performed on FC 500 flow cytometer (Beckman Coulter, FL, USA).

Conventional cytogenetics was done on 25 cells using Reverse banding using Heat and Giemsa (RHG) marking technique (300–400 bands). Molecular cytogenetic analysis (FISH) was performed on 200 cells using B-ALL probes including Vysis LSI ETV6(TEL)/RUNX1(AML1) ES dual-color translocation probe (Abbott, Chicago. USA).

## Results

Clinical presentation and laboratory work-up are summarized in Table 1.

**Table 1:** Clinical and laboratory features of the B-ALL case

Features	Case
Age (years)/gender	8/male
Symptoms/lymphadenopathy and organomegaly	Hemorrhagic syndrome with multiple ecchymoses stretching on all four limbs and the face with petechiae/absent
Complete blood cell count (CBC) data	
Hemoglobin	10 g/dl
WBC	20.3 × 10^9^/L
Platelets	18 × 10^9^/L
Peripheral blood smears examination	Showed significant number of blasts: 25%
Bone marrow examination data	
% of blasts	72%
Cytochemical MPO	Negative
Immunophenotypic data	
% of blasts	70%
Expression of antigens	
CD34	90%
HLA-DR	79%
CD13	0%
CD33	2%
MPO	1%
CD19	97%
CD10	97%
CD22	67%
CD79a	8%
IgMc	0%
cCD3	0%
sCD3	0%
CD7	0%
Conventional cytogenetics	Did not reveal any clonal chromosomal abnormalities
Cytogenetic study: fluorescence *in situ* hybridization (FISH)	Intrachromosomal amplification of chromosome 21 (iAMP21)

WBC: White Blood Cell; L: liter; g: gram; dL: deciliter; MPO: Myeloperoxidase; CD: cluster of differentiation; IgMc: cytoplasmic immunoglobulin M; c: cytoplasmic; s: surface

## Discussion

Acute leukemias are a diverse group of malignancies with a diverse range of clinical presentations, prognosis, and treatment protocols. Among them is acute lymphoblastic leukemia (ALL), which is a malignant tumor of the immature lymphoid cells. ALL is known to be the most common childhood cancer, and is responsible for about 80% of pediatric acute leukemia. Moreover, ALL is considered as the most frequent causes of death from cancer before 20 years of age [7, 8].

Accurate diagnosis in a short time is crucial for the successful clinical management of ALL. The use of morphological characterization of lymphoblast cells is not always easy. In some situations, these lymphoblasts cells are hardly distinguishable from myeloblast in many acute myeloid leukemia (AML) cases (undifferentiated acute myeloid leukemia, for example).

The development of monoclonal antibodies and the rapid evolution of lasers and analysis software have allowed flow cytometry (FC) to become a powerful tool for multiparametric cellular analysis over the last few decades. In 2001, the World Health Organization (WHO) integrated immunophenotyping of acute leukemia results into their proposed classification of malignancies of hematopoietic and lymphoid tissues. Since then, the use of FC in the diagnosis of hematological malignancies has considerably evolved the clinical management of acute leukemia. This integration made flow cytometry a critical step in the advancement of diagnosis of acute leukemia and an unavoidable stage to perform further subclassification. Moreover, immunophenotyping of AL by FC has become a key tool for detecting aberrant antigenic profiles that may prove useful for disease monitoring [9]. The WHO 2016 updated classification of hematological malignancies of myeloid neoplasms and acute leukemia has defined the criteria for assignment of the B-ALL lineage by FC on the basis of the intensity of expression of four key markers: CD19, CD79a, cytoplasmic CD22, and CD10 [10, 11]. According to the EGIL scoring system, CD79a and CD22 are the most critical markers in B-lineage assessment. They have a high-scoring value, with 2 points for each of them, while the other two markers, CD19 and CD10, came in second place with 1 point for each of them [6].

CD79a is expressed in nearly all B-cell development stages from pro-B to mature B cells, and decreases in secretory B cells [12]. As a result, CD79a can be used as an excellent pan cell marker for B-lineage assignment. According to the EuroFlow Consortium’s work, the use of CD79a is preferred over CD22 when it comes to B-lineage assessment because CD22 is not lineage-specific as it is also expressed at high levels in normal basophils, mast cells, and some dendritic cells [13]. For these reasons, any case of decrease in or lack of CD79a expression makes the diagnosis of B-ALL very challenging, especially in laboratories in developing countries, where flow cytometry analyses are not always available to help in diagnosis of AL and, when they are, they are limited in the number of markers used for lineage assignment. This limitation can easily lead to an incorrect diagnosis.

In our laboratory, for B-ALL lineage assignment, we used a comprehensive panel that included, in addition to CD79a, CD19, CD10, and CD22 recommended by the WHO, a cytoplasmatic and surface IgM. In this reported case, even though CD79a expression was severely low (CD79a, 8%), we were able to make the diagnosis of B-ALL on the basis of the expression of the other B markers included in the panel (CD19, 97%; CD10, 97%; and CD22, 67%) and, luckily, none of the other myeloid or T-cell markers were positive in this case. It has already been reported that the expression of CD79a in hematological cells is affected by the specificities of the clone CD79a used. As a result, many cases of aberrant expression of CD79a in other myeloid and lymphoid tumors have been reported [14–16]. However, this is not the situation in our case because we are not dealing with aberrant expression or nonspecific binding of CD79a markers that may vary by changing the clone used to detect CD79a expression in cells; instead, we are discussing the unusual lack of expression of this marker in B-lineage cells associated with this genetic abnormality, and no other similar cases in the literature have been reported before.

To meet the WHO’s requirements, we sent this bone marrow to another independent laboratory (French specialized laboratory) to perform the cytogenetic study. We also requested for an immunophenotyping of acute leukemia retest with a larger B-ALL panel. The goal is to get a wider antigenic profile for this case and to exclude the eventuality of a lack of expression of CD79a in B cells due to the specificity of the clone CD79a that we use in our laboratory. The obtained results from this laboratory show that we are dealing with a B-ALL case (B-II/B-III) with a lack of expression of CD79a.

The obtained results of the conventional cytogenetic study did not reveal any clonal chromosomal abnormalities. Therefore, we proceeded to the fluorescence *in situ* hybridization (FISH) study using commercially available LSI ETV6/RUNX1 ES dual-color translocation probe on 200 cells. The obtained results reveal the existence of intrachromosomal amplification of chromosome 21 (iAMP21). iAMP21 is a rare genetic abnormality that occurs in about 2–5% of pediatric patients with B acute lymphoblastic leukemia [17]. It leads to an increase in the number of copies (amplification) of the RUNX1 gene, at least three more copies, on chromosome 21. These types of genetic abnormalities are more often found in older children (> 5 years old) who suffer from B-ALL. In a previous study, Harrison *et al.* found that the median age of patients who have this abnormality is 9 years [18], which is close to the age of our patient (8 years), and the general data regarding the iAMP21 genetic abnormality are quite similar to our patient’s data, with a lower WBC count (< 50.10^9^/L) [19], blasts presented in most cases with a common/pre B phenotype , dim or dim partial expression of CD45, and generally no expression of myeloid lineage markers [20]. Studies have recognized iAMP21 as one of the most important anomalies recently discovered in ALL, in which genotype has a direct impact on treatment [17]. Consequently, the World Health Organization (WHO) has included this genetic abnormality in their revised 2016 classification of malignancies of hematopoietic and lymphoid tissues under new provisional heading called leukemia/B lymphoblastic lymphoma with iAMP21 [10].

## Conclusion

CD79a is one of the critical markers in B-ALL assignment. In our case, we have the chance to be assisted by many other markers of the B lineage that were positive in this case, and none of the other T or myeloid markers were positive. Also, it is necessary to mention the importance of proceeding to cytogenetic study, which in our case helped us to confirm the diagnosis made by flow cytometry by highlighting a cytogenetic abnormality that is specific to the B-ALL lineage.

## Data Availability

In accordance with local laws and regulations, any materials and de-identified data that are reasonably requested by others will be made available in a timely fashion.

## Abbreviations

AL: Acute leukemia
ALL: Acute lymphoblastic leukemia
AML: Acute myeloid leukemia
B-ALL: B acute lymphoblastic leukemia
BCR: B-cell receptor
BMA: Bone marrow aspirate
CD: Cluster of differentiation
EGIL: European Group for the Immunological Characterization of Acute Leukemias
FCI: Flow cytometric immunophenotyping
FC: Flow cytometry
FISH: Fluorescence *in situ* hybridization
HLA: Human leukocyte antigen
iAMP21: Intrachromosomal amplification of chromosome 21
IgM: Immunoglobulin M
sIg: Surface immunoglobulin
MPO: Myeloperoxidase
WBC: White blood cells
WHO: World Health Organization

## References

[1] Chu PG, Arber DA. CD79: a review. Appl Immunohistochem Mol Morphol. 2001;9(2):97–106. 10.1097/00129039-200106000-00001. 11396639

[2] Huang X, Takata K, Sato Y,* et al*. Downregulation of the B-cell receptor signaling component CD79b in plasma cell myeloma: a possible post transcriptional regulation. Pathol Int. 2011;61(3):122–9. 10.1111/j.1440-1827.2010.02634. 21355953

[3] Yu J-H, Dong J-T, Jia Y-Q,* et al*. Individualized leukemia cell-population profiles in common B-cell acute lymphoblastic leukemia patients. Chin J Cancer. 2013;32(4):213–23. 10.5732/cjc.012.10041. PMC3845576 22776235

[4] Mason DY, Cordell JL, Brown MH,* et al*. CD79a: a novel marker for B-cell neoplasms in routinely processed tissue samples. Blood. 1995;86(4):1453–9. 7632952

[5] Kozlov I, Beason K, Yu C, Hughson M. CD79a expression in acute myeloid leukemia t(8;21) and the importance of cytogenetics in the diagnosis of leukemias with immunophenotypic ambiguity. Cancer Genet Cytogenet. 2005;163(1):62–7. 10.1016/j.cancergencyto.2005.06.002. 16271957

[6] Bene MC, Castoldi G, Knapp W,* et al*. Proposals for the immunological classification of acute leukemias. European Group for the Immunological Characterization of Leukemias (EGIL). Leukemia. 1995;9(10):1783–6. 7564526

[7] DiGiuseppe JA. Acute lymphoblastic leukemia: diagnosis and detection of minimal residual disease following therapy. Clin Lab Med. 2007;27(3):533–49. 10.1016/j.cll.2007.05.005. 17658406

[8] Hunger SP, Mullighan CG. Acute lymphoblastic leukemia in children. Longo DL, ed. N Engl J Med. 2015;373(16): 1541–1552. 10.1056/nejmra1400972. 26465987

[9] Gupta N, Pawar R, Banerjee S,* et al*. Spectrum and immunophenotypic profile of acute leukemia: a tertiary center flow cytometry experience. Mediter J Hematol Infect Dis. 2019. 10.4084/MJHID.2019.017. PMC6402547 30858955

[10] Arber DA, Orazi A, Hasserjian R,* et al*. The 2016 revision to the World Health Organization classification of myeloid neoplasms and acute leukemia. Blood. 2016;127(20):2391–405. 10.1182/blood-2016-03-643544. 27069254

[11] Dworzak MN, Buldini B, Gaipa G,* et al*. AIEOP-BFM consensus guidelines 2016 for flow cytometric immunophenotyping of pediatric acute lymphoblastic leukemia. Cytometry B Clin Cytom. 2018;94(1):82–93. 10.1002/cyto.b.21518. 28187514

[12] Lai R, Juco J, Lee SF, Nahirniak S, Etches WS. Flow cytometric detection of CD79a expression in T-cell acute lymphoblastic leukemias. Am J Clin Pathol. 2000;113(6):823–30. 10.1309/391r-93yf-db4d-1l35. 10874883

[13] van Dongen JJM, Lhermitte L, Böttcher S,* et al*. EuroFlow antibody panels for standardized *n*-dimensional flow cytometric immunophenotyping of normal, reactive and malignant leukocytes. Leukemia. 2012;26(9):1908–75. 10.1038/leu.2012.120. PMC3437410 22552007

[14] Luger D, Yang Y-A, Raviv A,* et al*. Expression of the B-cell receptor component CD79a on immature myeloid cells contributes to their tumor promoting effects. PLoS ONE. 2013;8(10): e76115. 10.1371/journal.pone.0076115. PMC3797715 24146823

[15] Bo W, Mei K, Zhaoming W, Hongtian Y, Yanfeng B, Xiaoying N. CD79a positive T cell lymphoma with bone marrow involvement. Pathology. 2011;43(7):737–40. 10.1097/PAT.0b013e32834c726f. 22124260

[16] Tiacci E, Orvietani P-L, Bigerna B,* et al*. Tumor protein D52 (TPD52): a novel B-cell/plasma-cell molecule with unique expression pattern and Ca(2+)-dependent association with annexin VI. Blood. 2005;105(7):2812–20. 10.1182/blood-2004-07-2630. 15576473

[17] Garcia DRN, Arancibia AM, Ribeiro RC, Land MGP, Silva MLM. Intrachromosomal amplification of chromosome 21 (iAMP21) detected by ETV6/RUNX1 FISH screening in childhood acute lymphoblastic leukemia: a case report. Rev Bras Hematol Hemoter. 2013;35(5):369–71. 10.5581/1516-8484.20130111. PMC3832320 24255623

[18] Harrison CJ, Moorman AV, Schwab C,* et al*. An international study of intrachromosomal amplification of chromosome 21 (iAMP21): cytogenetic characterization and outcome. Leukemia. 2014;28(5):1015–21. 10.1038/leu.2013.317. PMC4283797 24166298

[19] Heerema NA, Carroll AJ, Devidas M,* et al*. Intrachromosomal amplification of chromosome 21 is associated with inferior outcomes in children with acute lymphoblastic leukemia treated in contemporary standard-risk children’s oncology group studies: a report from the children’s oncology group. J Clin Oncol. 2013;31(27):3397–402. 10.1200/JCO.2013.49.1308. PMC3770866 23940221

[20] Johnson RC, Weinberg OK, Cascio MJ,* et al*. Cytogenetic variation of B-lymphoblastic leukemia with intrachromosomal amplification of chromosome 21 (iAMP21). Am J Clin Pathol. 2015;144(1):103–12. 10.1309/AJCPLUYF11HQBYRB. 26071468

